# Metagenomics for the microbiological diagnosis of hospital-acquired pneumonia and ventilator-associated pneumonia (HAP/VAP) in intensive care unit (ICU): a proof-of-concept study

**DOI:** 10.1186/s12931-023-02597-x

**Published:** 2023-11-15

**Authors:** Morgane Heitz, Albrice Levrat, Vladimir Lazarevic, Olivier Barraud, Stéphane Bland, Emmanuelle Santiago-Allexant, Karen Louis, Jacques Schrenzel, Sébastien Hauser

**Affiliations:** 1 Intensive Care Unit, Annecy-Genevois Hospital, Site d’Annecy, 1 Avenue de L’hôpital, 74370 Metz Tessy, France; 2 Genomic Research Laboratory, Geneva University Hospitals and Faculty of Medicine, University of Geneva, Geneva, Switzerland; 3 Bacteriology Laboratory, Annecy-Genevois Hospital, Metz Tessy, France; 4 bioMérieux Marcy L’Etoile, 376 Chemin de l’Orme, 69280 Marcy l’Etoile, France; 5 BIOASTER Microbiology Technology Institute, 40 Avenue Tony Garnier, 69007 Lyon, France; 6 Bacteriology Laboratory, Geneva University Hospitals and Faculty of Medicine, University of Geneva, Geneva, Switzerland; 7 bioMérieux Grenoble, Centre Christophe Mérieux, 5 Rue Des Berges, 38024 Grenoble Cedex 01, France

**Keywords:** Metagenomics, Next generation sequencing (NGS), Hospital-acquired pneumonia, Ventilator-associated pneumonia, Microbiological diagnosis

## Abstract

**Background:**

Hospital-acquired and ventilator-associated-pneumonia (HAP/VAP) are one of the most prevalent health-care associated infections in the intensive care unit (ICU). Culture-independent methods were therefore developed to provide faster route to diagnosis and treatment. Among these, metagenomic next-generation sequencing (mNGS) has shown considerable promise.

**Methods:**

This proof-of-concept study describes the technical feasibility and evaluates the clinical validity of the mNGS for the detection and characterization of the etiologic agents causing hospital-acquired and ventilator-associated pneumonia. We performed a prospective study of all patients with HAP/VAP hospitalized in our intensive care unit for whom a bronchoalveolar lavage (BAL) was performed between July 2017 and November 2018. We compared BAL fluid culture and mNGS results of these patients.

**Results:**

A total of 32 BAL fluids were fully analyzed. Of these, 22 (69%) were positive by culture and all pathogens identified were also reported by mNGS. Among the culture-positive BAL samples, additional bacterial species were revealed by mNGS for 12 patients, raising the issue of their pathogenic role (colonization versus coinfection). Among BALF with culture-negative test, 5 were positive in mNGS test.

**Conclusions:**

This study revealed concordant results for pneumonia panel pathogens between mNGS and culture-positive tests and identified additional pathogens potentially implicated in pneumonia without etiologic diagnosis by culture. mNGS has emerged as a promising methodology for infectious disease diagnoses to support conventional methods. Prospective studies with real-time mNGS are warranted to examine the impact on antimicrobial decision-making and clinical outcome.

**Supplementary Information:**

The online version contains supplementary material available at 10.1186/s12931-023-02597-x.

## Background

Hospital-acquired and ventilator-associated-pneumonia (HAP/VAP) are one of the most prevalent health-care associated infections in the intensive care unit (ICU). HAP and, most prominently, VAP are associated with poor outcome and the overall attributable mortality of HAP/VAP is around 13% [1]. Critical care physicians are expected to provide a treatment adapted to causative agents and their antibiotic susceptibility profiles as early as possible in the course of the infection [2–4]. These data have been confirmed by the recent COVID-19 pandemic, many patients experienced bacterial pulmonary infection during the ICU stay [5]. International guidelines for the HAP/VAP management published in 2017 [6] pointed to potential benefits of narrow-spectrum antimicrobials in reducing the risk of toxicity and later emergence of microbial multiresistance. The HAP/VAP diagnosis is established when pneumonia occurs after ≥ 48 h of hospitalization and not incubating at the time of ICU admission. The time of onset of nosocomial pneumonia affects the etiology, and late onset is strongly associated with infection by multi-drugs-resistants pathogens. In this study, early- and late-onset VAP are defined as occurring within the first 5 and > 5 days of hospitalization, respectively [6, 7].

Conventional culture of a respiratory sample enables bacterial identification, quantification and antimicrobial susceptibility testing, which remains the gold standard to diagnose and treat lung infections. However, culture-based tests are time-consuming (48–72 h) and could lack sensitivity, particularly for slow-growing bacteria or when respiratory samples are collected after administration of an antibiotic therapy. Culture-independent tests for respiratory infections were developed to provide results within a few hours. These methods are usually based on PCR panels that detect only nucleic acids from common respiratory pathogens as well as selected antibiotic resistance genes (ARG) [8–11]. Metagenomic next-generation sequencing (mNGS) is a more recent methodology that refers to the concept of sequencing all the DNA of a sample to identify microorganisms but also their genomic traits like ARG, virulence factors or typing markers. Clinical applications of mNGS are currently drawing the attention of infectious diseases specialists and guidelines for mNGS-based diagnostics have emerged [12, 13]. Before launching clinical impact studies in the context of nosocomial pneumonia, it is of importance to evaluate the performance of mNGS on respiratory samples. We performed a proof-of-concept study to describe the technical feasibility of mNGS on respiratory samples and to evaluate its clinical relevance in detecting and characterizing causative agents of HAP/VAP.

## Methods

We conducted a monocentric prospective study that enrolled consecutive ICU patients of Annecy-Genevois Hospital (Metz-Tessy, France) who developed a HAP/VAP between July 2017 and November 2018. Pneumonia was diagnosed based on clinical features and compatible radiological imaging. An adjudication committee composed of independent experts reviewed all clinical, radiological and microbiological documents, to retrospectively confirm or deny the diagnosis of HAP/VAP suspected at the time of inclusion.

Each patient could be included in the study several times during ICU stay, whenever a new HAP/VAP episode was suspected. Patients aged under 18, immunosuppressed, with cystic fibrosis, under guardianship or pregnant were excluded. Patient information, including comorbidities, diagnosis at admission, current or recent antimicrobial therapy were collected from electronic charts.

The study protocol was approved by the French institutional ethics committee (CPP Sud-Méditerranée, Marseille, 2017-A00253-50, FRANCE). All patients or their relatives received an information letter and a non-objection form for participation was signed.

Whenever a HAP/VAP diagnosis was suspected, experienced physicians collected a BAL sample with a single-use flexible bronchoscope. Collected fluids were immediately sent to the local microbiology laboratory to perform cultures for diagnostic purpose. Whenever available, 1.2 mL of the BAL leftover was sent to BIOASTER institute (Lyon, France) for metagenomic sequencing within 48 h, while maintaining the cold chain (maximum 4 °C). mNGS data are then analyzed at Christophe Mérieux Center (bioMérieux-Grenoble, France).

### Conventional culture

BAL fluids were routinely cultured quantitatively on liquid and solid media and were examined daily for 72 h. Bacterial colonies were identified using matrix-assisted laser desorption ionization-time of flight mass spectrometry (MALDI-TOF MS, MALDI Biotyper®; reference library 2016 and 2017) [14]. Significance threshold was defined as ≥ 10^4^ colony forming units (CFU) per mL, counts below this cutoff were considered negative.

### Metagenomic next-generation sequencing (mNGS): sample processing and bioinformatics pipeline

**Fig. 1:**
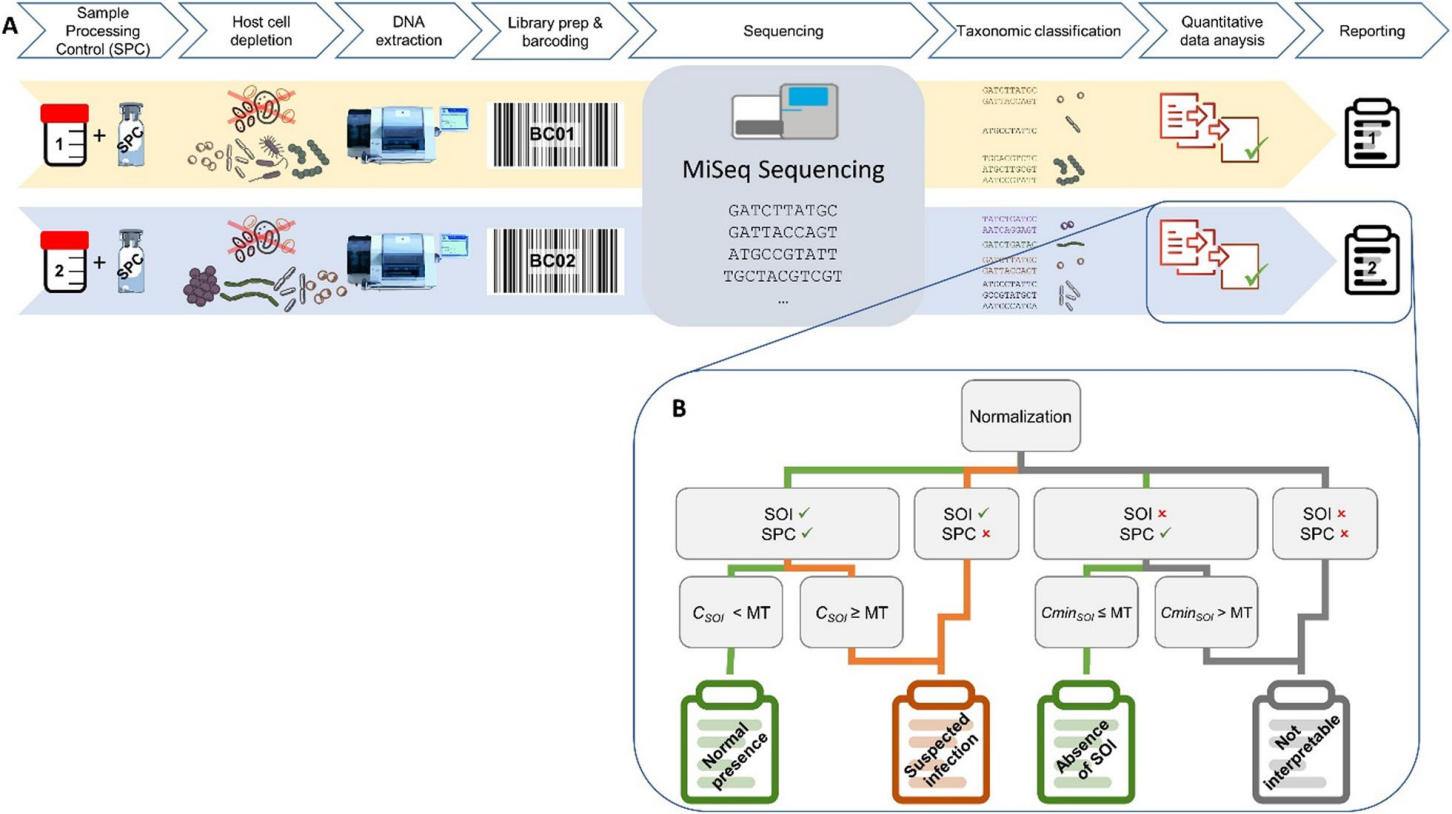
Complete workflow for clinical metagenomic analysis of BAL samples. **A** is the experimental workflow in which two independent samples are analyzed in the same sequencing run. **B** represents the rule of interpretation applied independently to each SOI to determine whether it is involved in patient infection or presence at normal concentration or absence in the sample or the inability to interpret the result. *SOI* species of interest, *SPC* sample processing control, *MT* metagenomic threshold

The complete clinical metagenomics workflow is described in Hauser et al. [15] and shown schematically in Fig. 1.

Briefly, to control the whole mNGS process, and to calculate the absolute concentration of the detected pathogen(s), a sample processing control (SPC) made with a defined amount of *Bacillus subtilis* (ATCC 19659 BioBall MultiShot 10E8 bioMérieux) was added to each BAL sample, before further processing. The final concentration of added *B. subtilis* within each sample was 1.7 × 10^4^ CFU/mL, close to the clinical significance threshold (10^4^ CFU/mL).

Bacterial DNA enrichment was performed using selective lysis of human cells with saponin and DNase I treatment to degrade the DNA released. The DNase I treatment also eliminates the extracellular DNAs and the DNAs of dead bacterial cells [16], thus favoring the detection of DNA originating from viable bacterial cells. After DNase inactivation, bacteria were disrupted by bead beating. Nucleic acids were extracted on an EasyMag® platform (bioMérieux) using the generic protocol (V2.0.1) and stored at − 20 °C.

Sequencing libraries were prepared with an optimized protocol of the Nextera® XT DNA Library Preparation Kit (Illumina).

The sequence data of each sample were analyzed with the bioinformatics pipeline described by Tournoud et al. [17] to quantify bacterial species. Briefly, reads were trimmed and filtered based on quality. Taxonomic read binning was performed with Kraken [18] using an internal reference database including sequences from (a) more than 10,000 genomes from the 19 species of interest (SOI)(common VAP causing pathogens listed in Table 1), (b) *B. subtilis* (used as SPC), (c) bacteria typically found in the human lung and oral cavity and (d) the human genome (*Homo sapiens* genome assembly GRCh37). Bacterial genome sequences were from both public (e.g. PATRIC [19], RefSeq [20], FDA ARGOS [21] and private (strains sequenced from BAL samples at bioMérieux) databases. [22]. Reads associated to a SOI with an average genome coverage depth > 1 × were assembled using the IDBA-UD 1.1.1 assembler.

**Table 1:** Microorganisms of the mNGS pneumonia panel (n = 19)

*Acinetobacter baumannii*
*Citrobacter freundii* *Citrobacter koseri*
*Enterobacter aerogenes*
*Escherichia coli*
*Haemophilus influenzae*
*Hafnia alvei*
*Klebsiella oxytoca*
*Klebsiella pneumoniae*
*Legionella pneumophila*
*Morganella morganii*
*Proteus mirabilis* *Proteus vulgaris*
*Providencia stuartii* *Pseudomonas aeruginosa*
*Serratia marcescens*
*Staphylococcus aureus*
*Stenotrophomonas maltophilia*
*Streptococcus pneumoniae*

To avoid spurious pathogen detection due to erroneous taxonomic assignments by Kraken, the assemblies were aligned with BLAST [23] against the pneumonia panel marker database built by selecting clade-specific MetaPhlAn2 [24] and 16S rDNA markers (for *Hafnia alvei*, *Proteus vulgaris* and *Morganella morganii*, for which no MetaPhlAn2 markers were available). The following rules were applied: (i) When at least one marker of the tested SOI is detected, taxonomic assignment of the sequences to the SOI was confirmed; (ii) When a marker from another species was detected and not the one of the tested SOI, the tested SOI was invalidated to avoid false positive detections induced by miss-classification of the reads. When no pathogen marker was detected, mainly because of low-coverage assemblies, no confirmation or invalidation of taxonomic classification to SOI were reported.

The metagenomics analysis report contained two sections. The first is focused on the 19 pneumonia panel pathogens. The results of each of these species of interest (SOI) are analyzed independently with 4 steps described in Fig. 1B:

i. First, the number of reads associated to each SOI and SPC are counted and normalized per million of bacterial reads. These normalized numbers of reads are then compared to a detection threshold, specific to each species, to determine if SOI and SPC can be considered detected;
ii. The second step consists in the calculation of the absolute concentration of genome equivalent (GEq) of the SOI (*C*_*SOI*_) or minimal detectable concentration of genome equivalent of SOI (*Cmin*_*SOI*_) based on the known SPC concentration (≈ 1.7 × 10^4^ GEq/mL) and the genome size of SOI and SPC. As the link between the concentration of GEq and the colony forming unit has not been established for each SOI, we used a unique metagenomic threshold (*MT*) of 5.3 × 10^3^ GEq/mL, defined as the concentration in genome equivalent of SOI above which infection can be suspected [15];
iii. In the third step, calculated *C*_*SOI*_ or *Cmin*_*SOI*_ are compared to *MT*;
iv. The last step consists in reporting the results of detection.

The rules of interpretation (Fig. 1B) define 4 possible outcomes:

i. *Suspected colonization*, when a SOI is detected and quantified below *metagenomics threshold (MT 5.3* × *10*^*3*^* GEq/mL)*;
ii. *Suspected infection*, when a SOI is detected and quantified at or above *MT* or when SOI is detected but not the SPC;
iii. *Absence of SOI detection*, when the SOI is not detected and the calculated *Cmin*_*SOI*_ is < *MT*;
iv. *Not interpretable*, when both SOI and SPC are not detected or when calculated *Cmin*_*SOI*_ is > *MT*.

The second part of the report provided the list of the 25 species with the highest read counts. In this analysis, the concentrations in GEq/mL are calculated without correction for genome size and validation filters are not applied. Therefore, this list provides an indication of the patient’s respiratory microbiota and may suggest an atypical infection by an organism not belonging to the pneumonia panel.

### Statistical analysis

Continuous demographic and clinical characteristics of patients are presented as median (range). Qualitative parameters are reported as percentages. The performance of mNGS test was analyzed with sensitivity, specificity, positive predictive value and negative predictive value, culture being used as the reference.

## Results

**Fig. 2:**
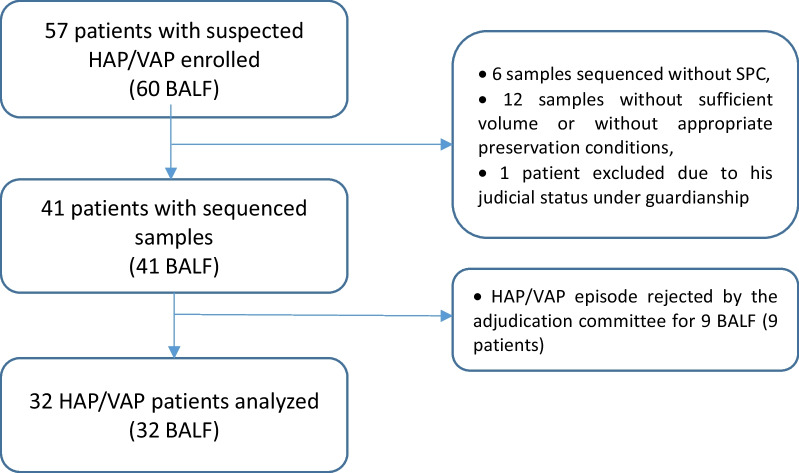
Flow chart

From July 2017 to November 2018, we enrolled 57 patients with HAP/VAP suspicion in ICU of Annecy-Genevois Hospital. We collected 60 BAL samples; among them, 41 (68%) were properly stored and had a sufficient volume to perform mNGS. After retrospective analysis by the adjudication committee, 32 of these 41 samples were linked to a confirmed HAP/VAP episode and were therefore selected for the final analysis (Fig. 2).

The patients characteristics are summarized in Table 2.

**Table 2:** Patients characteristics

	Patients, n (%)
Gender	
Male	21 (66)
Lifestyle	
Tobacco usage	7 (22)
Co-morbidities	
COPD	2 (6)
Lung cancer	2 (6)
Cardio-vascular disease	16 (50)
Admission diagnosis	
Trauma	10 (31)
Surgical	8 (25)
Medical	14 (44)
HAP/VAP	
Early-onset	16 (50)
Late-onset	16 (50)
Supportive care	
ARDS	13 (41)
Vasopressor drugs	18 (56)
RRT	2 (6)
Antibiotics	
BAL carried out under antibiotic treatment	12 (38)

*COPD* chronic obstructive pulmonary disease, *ARDS* acute respiratory distress syndrome, *RRT* renal replacement therapy

### Results from BAL culture (gold standard method)

22/32 BALF were positive by culture (69%), 7 with only one bacterial species ≥ 10^4^ CFU/mL and 15 with several micro-organisms (Table 3). *S. pneumoniae*, *S. aureus*, *E. coli* and *H. influenzae* were the most common species responsible for early-onset pneumonia; *E. coli*, *S. aureus* and *S. marcescens* were the main species in late-onset VAP.

**Table 3:** Comparison of the results of culture and mNGS in BALF positive in culture (n = 22)

BAL ID	SOI concordant in 2 methods	Additional bacteria detected in mNGS	Concomitant or previous antibiotic therapy
Concordant diagnosis between mNGS and culture (true positive) (n = 21)			
Fully concordant samples (n = 9)			
BAL 12	*Staphylococcus aureus*		No
BAL 22	*Escherichia coli* *Neisseria* spp.		No
BAL 32	*Pseudomonas aeruginosa* *Moraxella catarrhalis*		No
BAL 44	*Streptococcus pneumoniae* *Haemophilus influenzae* *Neisseria meningitidis*		No
BAL 49	*Escherichia coli*		Yes (piperacillin-tazobactam)
BAL 53	*Escherichia coli* *Enterobacter aerogenes*		No
BAL 55	*Streptococcus pneumoniae*		No
BAL 56	*Stenotrophomonas maltophilia*		Yes (piperacillin-tazobactam)
BAL 57	*Streptococcus pneumoniae* *Moraxella catarrhalis*		No
Concordant samples with additional bacteria detected (n = 12)			
BAL 8	*Escherichia coli*	*Haemophilus influenzae* *Streptococcus* spp.	No
BAL 13	*Escherichia coli*	*Akkermansia muciniphila* *Parabacteroides distasonis*	Yes (amoxicillin-clavulanic acid)
BAL 26	*Haemophilus influenzae* *Streptococcus agalactiae*	*Prevotella* spp.	No
BAL 29	*Staphylococcus aureus*	*Escherichia coli* *Ralstonia pickettii*	Yes (ceftriaxone, levofloxacin)
BAL 30	*Klebsiella pneumoniae* *Haemophilus influenzae*	*Moraxella catarrhalis* *Prevotella* spp. *Neisseria* spp.	No
BAL 31	*Serratia marcescens* *Enterococcus faecalis* *Streptococcus anginosus*	*Gardnerella vaginalis*	Yes (amoxicillin)
BAL 35	*Klebsiella oxytoca*	*Klebsiella pneumoniae* *Escherichia coli* *Enterobacter aerogenes* *Serratia marcescens* *Hafnia alvei* *Citrobacter freundii* *Proteus mirabilis* *Prevotella* spp. *Nesseria* spp.	No
BAL 38	*Pseudomonas aeruginosa* *Haemophilus influenzae* *Streptococcus pneumoniae* *Moraxella catarrhalis*	*Rothia mucilaginosa*	No
BAL 47	*Serratia marcescens*	*Bordetella bronchoseptica*	No
BAL 48	St*aphylococcus epidermidis*	*Streptococcus pneumoniae* *Rothia mucilaginosa*	No
BAL 52	*Staphylococcus aureus*	*Aggregatibacter aphrophilus*	No
BAL 60	*Haemophilus influenzae*	*Streptococcus constellatus*	No
False positive detection (n = 1)			
BAL 59	*Streptococcus anginosus*	*Haemophilus influenzae*	No

Only species identified by culture (present above the clinical threshold of 10^4^ CFU/mL) and/or those exceeding metagenomics threshold (5.3 × 10^3^ GEq/mL) in the mNGS analysis are presented

### Results from mNGS

The challenges of this study are threefold. First, confirming results from the reference method using NGS. Second, diagnosing potentially pathogenic bacteria that may have gone undetected in culture. Third, providing diagnoses for patients in cases where cultures failed to identify the pathogen.

### mNGS data of culture-positive BALF

In 9/22 culture-positive BALF, mNGS and culture results were fully concordant (Table 3). In 12/22 samples, mNGS confirmed the culture-based detections above the clinical threshold but revealed additional species, some of which belonged to pneumonia panel (BAL 8, 29, 35, 48). The only BALF in which culture-positivity for a pathogen (*S*. *aureus*) was not confirmed by mNGS was BAL 59. The number of reads assigned to this species was below the mNGS detection threshold defined for *S*. *aureus*, which did not allow reliable quantification of the pathogen load. The performance of mNGS was even higher when only pneumonia panel SOIs were analyzed. In this case, mNGS confirmed culture results for 17/22 BALFs. Three samples (BAL 8, 29, 35) had both true positive and false positive SOI detections, one sample (BAL 48) had a false positive SOI detection and in one sample (BAL 59) a false negative (*H. influenzae*) and a false positive SOI (*S.aureus*) were identified. The Additional file 1: Table S1 provides a more detailed breakdown of all the results (quantification, …).

### mNGS data of culture-negative BALFs

Among 10 BALF considered culture-negative (Table 4), 5 were mNGS-positive (BAL 20, 33, 34, 43, 45) for pulmonary pathogen like *E. coli* or *H. influenzae* and others species which do not belong to the classical pneumonia pathogen.

**Table 4:** Comparison of the results of culture and mNGS for BALF considered culture negative (n = 10)

BAL ID	SOI concordant in 2 methods but under threshold in culture	Additional bacteria detected in mNGS	Concomitant or previous antibiotic therapy
BAL 20		*Escherichia coli* *Streptococcus anginosus*	Yes (piperacillin–tazobactam then amoxicillin–clavulanic acid)
BAL 45	*Alpha-hemolytic streptococci*	*Haemophilus influenzae* *Streptococcus pneumoniae*^a^ *Streptococcus parasanguinis*^a^	Yes (cefotaxime and metronidazole)
BAL 23			No
BAL 33		*Ralstonia pickettii* *Lactobacillus oris*	No
BAL 34		*Ralstonia pickettii*	Yes (amoxicillin–clavulanic acid)
BAL 36			Yes (cefotaxime and metronidazole)
BAL 41			Yes (piperacillin–tazobactam)
BAL 42	*Staphylococcus warneri*		No
BAL 43		*Streptococcus thermophilus*	Yes (cefotaxime and metronidazole)
BAL 46			Yes (cefotaxime and metronidazole)

Only species identified by culture (present above or below the clinical threshold of 10^4^ CFU/mL) and/or those exceeding metagenomics threshold (5.3 × 10^3^ GEq/mL) in the mNGS analysis are presented

^a^Belong to alpha-hemolytic streptococci

### Quality control and sample processing control

In 23/32 samples SPC were detected. When detection failed, the negative identification of pathogens cannot be validated, sample calibration was not possible and quantification in GEq/mL of the bacteria was not achievable.

### Assignment error

In almost all samples *E. cloacae* and *M. tuberculosis* were detected above the metagenomics threshold. These identifications suggest a potential assignment error. Verification methods were applied to all samples, demonstrating the absence of detection of these bacteria. MetaPhlAn and BLAST tools used to confirm mNGS results did not validate the identification of *E. cloacae* in any sample.

### Time to results (mNGS)

In our study, the mean laboratory turnaround time was above 45 h: 6 h were needed for sample preparation and 39 h for Illumina sequencing. Duration of bioinformatics analysis and report generation steps depended on the number of reads generated for each sample and the number of threads available for data analysis. Typically, it ranged between 30 min and 6 h.

### Performance of mNGS in detecting SOI

The detection by mNGS of SOI likely to be the source of infection is compared with that of microbial culture for all 19 SOI in the 32 BALF samples (total data population = 608), using a 2 × 2 confusion matrix [25]. Results considered uninterpretable due to the absence of detection of both SOI and SPC (Fig. 1) were counted as negative detections and their quantities are indicated in brackets in Table 5. mNGS showed 96.2% sensitivity and a specificity of 97.8% (Table 5) using culture results as a reference. The positive and negative predictive value were 65.8% and 97.8%, respectively.

**Table 5:** Confusion matrix to compare the performance of mNGS against microbial culture for the detection of species of interest (SOI)

	Predicted condition by mNGS		
	Predicted positive C_SOI_ ≥ MT PP = 38	Predicted negative C_SOI_ < MT or no detection PN = 570	
Microbial culture condition			
Positive Detection ≥ CT P = 26	True positive TP = 25	False negative FN = 0 (1 without SPC detection)	Sensitivity $\mathrm{Sensitivity}=\frac{\mathrm{TP}}{\mathrm{P}}$ 96.2%
Negative (N) Detection < CT or no detection N = 582	False positive FP = 13	True negative TN = 269 (300 without SPC detection)	Specificity $\mathrm{Specificity}=\frac{\mathrm{TN}}{\mathrm{N}}$ 97.8%
	Positive predictive value $\mathrm{PPV}=\frac{\mathrm{TP}}{\mathrm{PP}}$ 65.8%	Negative predictive value $\mathrm{NPV}=\frac{\mathrm{TN}}{\mathrm{PN}}$ 99.8%	

19 SOI were assessed in 32 BALF representing a total data population equal to 608. Microbial cultures are classified as positive (P) when the SOI is quantified above or equal to the culture threshold (CT) and negative (N) when the SOI is quantified below the CT or when the SOI was not detected. Metagenomics predicts the result as positive (PP) when the concentration of SOI (C_SOI_) is calculated as equal to or greater than MT. Metagenomics predicts the result as negative (PN) when the C_SOI_ is calculated to be lower than MT. Results that cannot be interpreted by mNGS, i.e. those in which neither SOI nor SPC has been detected (Fig. 1), are considered negative and the quantities are indicated separately in brackets. True positive (TP) and true negative (TN) correspond respectively to the concordant positive or negative results between the 2 methods. False negative (FN) corresponds to positive results in microbial culture but predicted as negative by mNGS. False positive (FP) corresponds to negative results in microbial culture but predicted as positive by mNGS. Sensitivity represents the culture positive detection of SOI classified as positive by mNGS to the total number of positive culture detection. Specificity expresses the ratio of correctly predicted negative by mNGS to the total number of negative detections of SOI by culture. Positive predictive value (PPV), also called precision, represents the proportion of positive detections by culture that were correctly classified to the total number of positive predicted detection by mNGS. Negative predictive value (NPV), or true negative accuracy, measures the proportion of negative detection by culture that were correctly predicted as negative by mNGS to the total number of negative prediction

## Discussion

The purpose of this study was to evaluate the feasibility of mNGS for the direct detection of organisms in BAL fluid without using specific tests for different organisms. The recent COVID-19 pandemic and the occurrence of multiple bacterial superinfections complicating viral infection in ICU have underscored the significance of rapid diagnostics for tailoring treatments and avoiding the indiscriminate use of broad-spectrum antibiotics. While mNGS is not novel approach and has been explored by others, this study holds merit as it contributed to the currently limited knowledge in this field.

Our main encouraging result was that all respiratory pathogens identified by the culture were also detected by mNGS method. To be considered as a diagnosis test, it is essential that no false negative is detected when using a new method. 96% of species positive in culture exceeded the positivity threshold in the mNGS analysis. Only one false negative case is reported, the pathogen in question was detected by mNGS but the result was considered non interpretable due to an insufficient number of sequencing reads. The other main results of interest are the discrepancies between mNGS and culture results, mNGS helps identifying the pneumonia etiology in patients without microbiological diagnosis. In this study, in 7 patients, mNGS revealed putative HAP/VAP-causing agents that were either undetected or reported under the clinical significance threshold by culture tests. Some of these species were typical respiratory pathogens (e.g. *E*. *coli* and* H*. *influenzae)* while the others did not belong to the respiratory panel (e.g. *Prevotella* spp.*, Neisseria* spp. or *Gardnerella vaginalis*). The detection of microorganisms by mNGS can reflect normal microbiota, colonizers, infection-causing microorganisms or sample contamination. When mNGS is used to detect pathogens in normally sterile liquids, such as urine, cerebrospinal or synovial fluids, the assignment of clinical significance for detected organisms is more straightforward [26, 27]. For BALF samples, the interpretation is more complex because the presence of a microorganism does not necessarily reflect an infection. In five out of 10 VAP cases with sterile BALF or BALF with cultured bacteria present under the clinical significance threshold, several bacterial species were considered mNGS-positive (above the MT threshold). In two of these cases (BAL 20, 45), these species belonged to the pneumonia panel (*E*. *coli*, *H*. *influenzae*). But in two others samples without microbiological diagnosis, *R. pickettii* was found above the threshold. We cannot exclude the possibility of reagent contamination by *R*. *pickettii* DNA [28–30] or an infection. Species of the genus *Ralstonia* are recognized as emerging gram-negative nosocomial pathogens causing bacteremia, VAP and meningitis. In a mixed infection, one bacterial species could potentially promote the pathogenicity of another. While coinfections with several pathogens in the context of VAP have been described using culture tests [31], there is no consensus on whether in such situations bacteria found below the clinically significance thresholds (< 10^4^ CFU/mL) should be considered as pneumonia etiological agents. This is even less evident with mNGS that identifies a wider spectrum of bacteria per sample as compared to culture. For example, previous studies found *Mycoplasma* in many BALF from patients with VAP but these were mainly commensal species and not *M. pneumoniae* [31, 32]*.* This is a typical illustration of a bacterial taxon, not cultured with standard techniques but detected by mNGS, that could play a role in the pathogenicity of VAP [33]. It would be interesting to evaluate in a prospective clinical study the impact of real time mNGS results where the main objectives would be the clinical course of patients in order to determine the status of these newly diagnosed pathogens. One group could use the strategy of a prompt antibiotic de-escalation guided by mNGS results and the other the standard of care method of antibiotic adaptation. The impact of this technique on antibiotic utilization and antibiotic outcomes is unknown. It is possible that this method will lead to MORE antibiotic use rather than lest because more organisms are identified, some of which may may just be colonizers.

Twelve out of the 32 patients received antibiotics before BAL sampling: for 5 of them (BAL 13, 29, 31, 49, 56), pneumonia pathogens exceeded the clinical significance threshold in both culture and mNGS analyses. In other two cases (BAL 20, 45), bacteria detected by mNGS were (theoretically) susceptible to the previously administered antibiotics, which may explain their low abundance or absence in the culture. For example, the BAL 45 was mNGS-positive for *H*. *influenzae* and the BAL 20 for *E. coli* while the culture did not detect these pathogens, most likely because these patient received an efficient antibiotherapy on these bacteria before sampling (cefotaxime for patient 45 and piperacillin–tazobactam for patient 20).

The issue of the relative versus absolute quantification of microbiota members in respiratory samples assessed by culture-free methods was raised by Emonet et al. [34] and tackled in a metataxonomic (16S-targeted metagenomics) analysis of respiratory samples combined with panbacterial qPCR assay [31]. In our study, as suggested earlier [35], *B*. *subtilis* was added as a sample processing control (SPC) to BALF at a defined concentration to enable identification of possible analytical failures and extrapolate the absolute abundance of pathogens. Our study is one of the first to use SPC to provide quantitative mNGS data for respiratory pathogens, expressed as GEq/mL. However, the correlation between GEq/mL and CFU/mL has not yet been clearly established and its rather conservative interpretation remains to be prospectively validated. Currently, most published studies comparing culture and mNGS give raw mNGS results (detection or not). Specie detected by mNGS were considered positive without confirmation tests like MetaPhlAn and BLAST tools. Clinical interpretation is therefore difficult, and mNGS results can simply be regarded as a description of the lung microbiome with pathogen and non-pathogen species. No guidelines have been established to assist clinicians in interpreting positive mNGS results and the final clinical diagnosis was determined by several experienced clinicians, leading to unavoidable subjective biases due to the lack of unified standards [36, 37]. Some authors have established rules to mitigate subjectivity in the interpretation of mNGS results. For example, Langelier et al. classified microbes as confirmed pathogens if (1) both clinical testing and mNGS identified the microbe, (2) there was existing literature evidence of pathogenicity in the lungs, and (3) the score was as least twofold greater than that of any other microbe of the same type (virus, bacteria, or fungus) identified in the patient [38] and Jin et al. considered results positive when the relative abundance of pathogens detected by mNGS at the genus level was greater than or equal to 30% [39]. Our study is therefore one of the first to use an SPC to provide objective quantitative results and assist in interpretation using a pathogenicity threshold [40].

Currently, the scope of mNGS application in clinical microbiology is confined to situations where cultures fail to identify pathogens, especially in infections associated with (prior) usage of antibiotics. This is also applicable to identification of slow-growing or difficult-to-grow pathogens such as *M. tuberculosis*, *Legionella* spp., viruses or fungi; infections by these micro-organisms might be currently underestimated [26, 41].

This proof-of-concept study has several limitations. First, the application of mNGS was possible only for 41 BAL fluids among the 60 collected. Then, 9/41 (23%) were excluded due to pneumonia misdiagnosis. HAP/VAP diagnosis remains complex even for experts in the field; physicians may come to different conclusions about whether a given patient meets criteria for pneumonia especially due to difficulties in interpretation of chest radiographies in the ICU. Secondly, it does not provide antimicrobial susceptibility predictions thus limiting potential impact on antibiotic use. Thirdly, the mNGS process currently lacks standardization, complicating interpretation of results. One of the main preanalytical mNGS issues is that BAL samples contain many human cells. Therefore, removal of the overwhelming human DNA is critical for increasing the sensitivity of microbial signals in mNGS datasets [34]. An advantage of the mNGS pipeline used in our study is that combination of saponin and DNAse treatment remove extracellular DNA and DNA from damaged cells (dead bacteria), providing a clinically relevant bacterial detection. Bioinformatics analysis of mNGS data also constitutes a challenge. In our dataset, *E. cloacae* and *M. tuberculosis* were identified in nearly all samples above the mNGS threshold. However, MetaPhlAn and BLAST tools used to confirm results did not validate the identification of *E. cloacae* in any sample. These false identifications suggest that some bacterial genomic sequences from the reference database could be contaminated with those of human origin, resulting in misclassification of human reads as bacterial. *M. tuberculosis*, whose detection was not checked with BLAST or MetaPhlAn, could also be confused with other non-pathogenic mycobacteria. A second assessment was conducted using mNGS, utilizing Oxford Nanopore MinION and another database named WIMP. It does not find this *E. cloacae* and *M. tuberculosis* in any of the samples, thus confirming a potential error of assignation. Therefore, the quality of the reference database is of paramount importance for reliable taxonomic assignments and accurate pathogens detection. In our protocol, the turnaround time was 45–51 h, which included 39 h of Illumina MiSeq sequencing. Current sequencing technologies and mNGS pipelines allow an even faster time to result of < 30 h [42] with Illumina or ~ 6 h with Oxford Nanopore [43], allowing identification of pathogens in a timeframe that is compatible with the clinical practice. Reducing the time to pathogen identification, which was not the objective of our study, not only improves patient care but also contributes to a more rational use of antibiotics. But some might argue that this faster time to result is already achieved with the advent of PCR in clinical practice. However, in cases like the Filmarray Pneumonia Panel, implementing technical changes for new bacteria detection is time-consuming. To obtain authorization for clinical use, the process takes nearly a year. Additionally, the acid nucleic purification used in PCR employs a non-specific extraction of all acid nucleic (micro-organism and human DNA) using magnetic beads. To the opposite, the DNAse and the comparison of the size of sequenced genomes allows differentiation between dead and alive bacterial genomes, providing clinically relevant information.

This method is suitable in reference laboratory where the technical expertise and staffing required for performing mNGS are available. However, it is challenging to envision conducting these analyses in frontline hospital laboratories. Also, transporting samples to a reference laboratory could potentially increase the turn-around time, diminishing the method’s appeal. To avoid this, we could consider locally performing the technical processes of DNA extraction and libraries preparation, then transferring the data to bioinformatics reference laboratory with an on-call service to obtain results as quickly as possible.

In critically ill or severely immunocompromised patients, mNGS has the potential to improve clinical care and outcome by providing timely and efficient pathogen identification. Further studies are needed to determine the clinical impact of mNGS as an adjunct or even alternative to currently used diagnostic tests for HAP/VAP.

## Conclusion

We demonstrated technical feasibility and clinical proof-of-concept for the use of mNGS for diagnosing HAP/VAP in the ICU setting. Our results matched well those of culture tests and identified pathogens potentially implicated in pneumonia cases without etiologic diagnosis. Although the costs related to wet lab and bioinformatics resources currently limit the use of mNGS in clinical routine, this methodology is complementary to conventional approaches in antimicrobial decision-making in patients with infections, which may further improve clinical outcomes.

### Supplementary Information

**Additional file 1: Table S1.** Comparison of the results of bacterial quantification by culture and mNGS in 32 BALFs. Only species identified by culture (present above or below the clinical threshold of 104 CFU/mL) and/or those exceeding metagenomics threshold (5.3 × 103 GEq/mL) in the mNGS analysis are presented. In bold: pathogen detections above the clinical threshold for culture or above the mNGS positivity threshold. Species not belonging to the pneumonia panel are indicated between square brackets. Dotted lines indicate no detection. False positive (FP) and true positive (TP) SOI (pneumonia panel) are indicated for mNGS using culture data as a reference. NI, non-interpretable quantification of SOI; indicated only for SOIs identified by culture (below or above clinical threshold). FP* and NI*: corresponding SOIs were detected by culture under the clinical threshold. “ > MT” means that SOI was detected but SPC was undetected (see “Methods”).

## Data Availability

The datasets used and/or analyzed during the current study are available from the corresponding author on reasonable request.

## Abbreviations

ARDS: Acute respiratory distress syndrome
BAL: Bronchoalveolar lavage
BALF: Bronchoalveolar lavage fluid
CFU: Colony forming unit
COPD: Chronic obstructive pulmonary disease
CT: Culture threshold
DNA: Deoxyribonucleic acid
GEq: Genome equivalent
HAP: Hospital-acquired pneumonia
ICU: Intensive care unit
mNGS: metagenomics Next Generation Sequencing
MT: Metagenomic threshold
PCR: Polymerase Chain Reaction
RRT: Renal replacement therapy
SOI: Species of interest
SPC: Sample processing control
VAP: Ventilator associated pneumonia
