# Supplementary material for: Metagenomics for the microbiological diagnosis of hospital-acquired pneumonia and ventilator-associated pneumonia (HAP/VAP) in intensive care unit (ICU): a proof-of-concept study
Source: Respir Res. 2023 Nov 15;24:285. doi: 10.1186/s12931-023-02597-x (PMC10648381; doi:10.1186/s12931-023-02597-x)
Supplement: Supplementary file 1 — Additional file 1: Table S1. Comparison of the results of bacterial quantification by culture and mNGS in 32 BALFs. Only species identified by culture (present above or below the clinical threshold of 104 CFU/mL) and/or those exceeding metagenomics threshold (5.3 × 10^3^ GEq/mL) in the mNGS analysis are presented. In bold: pathogen detections above the clinical threshold for culture or above the mNGS positivity threshold. Species not belonging to the pneumonia panel are indicated between square brackets. Dotted lines indicate no detection. False positive (FP) and true positive (TP) SOI (pneumonia panel) are indicated for mNGS using culture data as a reference. NI, non-interpretable quantification of SOI; indicated only for SOIs identified by culture (below or above clinical threshold). FP* and NI*: corresponding SOIs were detected by culture under the clinical threshold. “ > MT” means that SOI was detected but SPC was undetected (see “Methods”). [file 12931_2023_2597_MOESM1_ESM.docx]

**Additional appendix**

Table S1: Comparison of the results of bacterial quantification by culture and mNGS in 32 BALFs

| **BAL ID** | **Culture (CFU/mL)** | **mNGS (GEq/mL)** | **Concomitant or previous antibiotic therapy** | **early-onset HAP/VAP** |
| --- | --- | --- | --- | --- |
| **BALF with VAP pathogens above the clinical significance in culture tests (n=22)** | | | |  |
| BAL 8 | ***Escherichia coli* (> 10^5^)**  *Haemophilus influenzae* (< 10^3^)  *Klebsiella oxytoca* (> 10^3^)  [ - - - - - - - - ] | ***Escherichia coli* (> MT)**  ***Haemophilus influenzae* (> MT)**  *Klebsiella oxytoca* (Not Interpretable)  **[*Streptococcus* spp.** **(> MT)]** | No | yes |
| BAL 12 | ***Staphylococcus aureus* (> 10^5^)** | ***Staphylococcus aureus* (8 x 10^4^)** | No | no |
| BAL 13 | ***Escherichia coli* (> 10^5^)**  [ - - - - - - - - ]  [ - - - - - - - - ] | ***Escherichia coli* (5 x 10^5^)**  **[*Akkermansia muciniphila* (9 x 10^3^)]**  **[*Parabacteroides distasonis* (7 x 10^3^)]** | Yes (amoxicillin-clavulanic acid) | yes |
| BAL 22 | ***Escherichia coli* (> 10^5^)**  **[*Neisseria* spp. (> 10^4^)]** | ***Escherichia coli* (> MT)**  **[*Neisseria* spp. (> MT)]** | No | no |
| BAL 26 | ***Haemophilus influenzae* (> 10^5^)**  *Klebsiella pneumoniae* (10^3^)  **[*Streptococcus agalactiae* (> 10^5^)]**  [ - - - - - - - - ]  [ - - - - - - - - ] | ***Haemophilus influenzae* (3 x 10^7^)**  *Klebsiella pneumoniae* (Not Interpretable)  **[*Streptococcus agalactiae* (2 x 10^5^)]**  **[*Streptococcus* spp. (> 10^6^)]**  **[*Prevotella* spp. (> 10^6^)]** | No | yes |
| BAL 29 | ***Staphylococcus aureus* (> 10^4^)**  - - - - - - - - - -  [ - - - - - - - - ] | ***Staphylococcus aureus* (5 x 10^5^)**  ***Escherichia coli* (10^4^)**  **[*Ralstonia pickettii* (5 x 10^4^)]** | Yes (ceftriaxone, levofloxacin) | yes |
| BAL 30 | ***Klebsiella pneumoniae* (> 10^5^)**  ***Haemophilus influenzae* (> 10^5^)**  [ - - - - - - - - ]  [ - - - - - - - - ]  [ - - - - - - - - ] | ***Klebsiella pneumoniae* (> MT)**  ***Haemophilus influenzae* (> MT)**  **[*Moraxella catarrhalis* (> MT)]**  **[*Prevotella* spp. (> MT)]**  **[*Neisseria* spp. (> MT)]** | No | no |
| BAL 31 | ***Serratia marcescens* (> 10^4^)**  **[*Enterococcus faecalis* (> 10^4^)]**  **[*Streptococcus anginosus* (10^5^)]**  [ - - - - - - - - ] | ***Serratia marcescens* (> MT)**  **[*Enterococcus faecalis* (> MT)]**  **[*Streptococcus anginosus* (> MT)]**  **[*Gardnerella vaginalis* (> MT)]** | Yes (amoxicillin) | no |
| BAL 32 | ***Pseudomonas aeruginosa* (> 10^5^)**  **[*Moraxella catarrhalis* (> 10^4^)]** | ***Pseudomonas aeruginosa* (2 x 10^6^)**  **[*Moraxella catarrhalis* (5 x 10^6^)]** | No | no |
| BAL 35 | - - - - - - - - - -  ***Klebsiella oxytoca* (> 10^5^)**  - - - - - - - - -  - - - - - - - - -  - - - - - - - - -  - - - - - - - - -  - - - - - - - - -  - - - - - - - - -  [ - - - - - - - - ]  [ - - - - - - - - ] | ***Klebsiella pneumoniae* (9 x 10^6^)**  ***Klebsiella oxytoca* (> 2 x 10^5^)**  ***Escherichia coli* (2 x 10^5^)**  ***Enterobacter aerogenes* (3 x 10^4^)**  ***Serratia marcescens* (10^4^)**  ***Hafnia alvei* (10^4^)**  ***Citrobacter freundii* (8 x 10^3^)**  ***Proteus mirabilis* (7x 10^3^)**  **[*Prevotella* spp. (> 10^5^)]**  **[*Nesseria* spp. (> 10^4^)]** | No | no |
| BAL 38 | ***Pseudomonas aeruginosa* (> 10^5^)**  ***Haemophilus influenzae* (> 10^5^)**  ***Streptococcus pneumoniae* (> 10^5^)**  **[*Moraxella catarrhalis* (> 10^5^)]**  [ - - - - - - - - ] | ***Pseudomonas aeruginosa* (> MT)**  ***Haemophilus influenzae* (> MT)**  ***Streptococcus pneumoniae* (> MT)**  **[*Moraxella catarrhalis* (> MT)]**  **[*Rothia mucilaginosa* (> MT)]** | No | yes |
| BAL 44 | ***Streptococcus pneumoniae* (> 10^5^)**  ***Haemophilus influenzae* (> 10^4^)**  **[*Neisseria meningitidis* (> 10^4^)]** | ***Streptococcus pneumoniae* (> MT)**  ***Haemophilus influenzae* (> MT)**  **[*Neisseria meningitidis* (> MT)]** | No | yes |
| BAL 47 | ***Serratia marcescens* (> 10^4^)**  [ - - - - - - - - ] | ***Serratia marcescens* (> MT)**  **[*Bordetella bronchoseptica* (> MT)]** | No | no |
| BAL 48 | - - - - - - - - - -  **[*Staphylococcus epidermidis* (> 10^4^)]**  [*Haemophilus parainfluenzae* (> 10^3^)]  [ - - - - - - - - ] | ***Streptococcus pneumoniae* (10^4^)**  **[*Staphylococcus epidermidis* (10^4^)]**  **[*Haemophilus parainfluenzae* (3 x 10^4^)]**  **[*Rothia mucilaginosa* (3 x 10^5^)]** | No | yes |
| BAL 49 | ***Escherichia coli* (> 10^5^)** | ***Escherichia coli* (2 x 10^7^)** | Yes (piperacillin-tazobactam) | no |
| BAL 52 | ***Staphylococcus aureus* (> 10^4^)**  *Escherichia coli* (< 10^3^)  [ - - - - - - - - ] | ***Staphylococcus aureus* (2 x 10^6^)**  *Escherichia coli* (2 x 10^2^)  **[*Aggregatibacter aphrophilus* (2 x 10^4^)]** | No | no |
| BAL 53 | ***Escherichia coli* (> 10^5^)**  ***Enterobacter aerogenes* (> 10^4^)** | ***Escherichia coli* (> MT)**  ***Enterobacter aerogenes* (> MT)** | No | no |
| BAL 55 | ***Streptococcus pneumoniae* (> 10^7^)** | ***Streptococcus pneumoniae* (> MT)** | No | yes |
| BAL 56 | ***Stenotrophomonas maltophilia* (> 10^4^)**  *Citrobacter freundii* (< 10^3^)  *Enterobacter cloacae* (> 10^3^) | ***Stenotrophomonas maltophilia* (9 x 10^4^)**  *Citrobacter freundii* (2 x 10^3^)  *Enterobacter cloacae* (5 x 10^3^) | Yes (piperacillin-tazobactam) | no |
| BAL 57 | ***Streptococcus pneumoniae* (> 10^4^)**  **[*Moraxella catarrhalis* (> 10^4^)]** | ***Streptococcus pneumoniae* (2 x 10^6^)**  **[*Moraxella catarrhalis* (10^7^)]** | No | no |
| BAL 59 | ***Staphylococcus aureus* (> 10^5^)**  *Haemophilus influenzae* (> 10^3^) **[*Streptococcus anginosus* (> 10^5^)]** | *Staphylococcus aureus* (Not Interpretable)  ***Haemophilus influenzae* (> MT)**  **[*Streptococcus anginosus* (> MT)]** | No | yes |
| BAL 60 | ***Haemophilus influenzae* (> 10^4^)**  [*Streptococcus constellatus* (10^3^)]  *Staphylococcus aureus* (> 10^3^) | ***Haemophilus influenzae* (3 x 10^4^)**  **[*Streptococcus constellatus* (2 x 10^4^)]**  *Staphylococcus aureus* (2 x 10^3^) | No | yes |
| ***BALF considered culture-negative (n=10)*** | | | |  |
| BAL 20^a^ | - - - - - - - -  *Klebsiella pneumoniae* ( 10^3^)  [ - - - - - - - - ] | ***Escherichia coli* (8 X 10^5^)**  *Klebsiella pneumoniae (*Not Interpretable*)*  **[*Streptococcus anginosus* (8 x 10^4^)]** | Yes (piperacillin-tazobactam then amoxicillin- clavulanic acid) | no |
| BAL 45^b^ | - - - - - - - - - -  alpha-hemolytic streptococci (< 10^4^) | ***Haemophilus influenzae* (7 x 10^3^)**  *Streptococcus pneumoniae* (2 x 10^3^)^a^  [*Streptococcus parasanguinis* (4 x 10^3^)]^a^ | Yes (cefotaxime and metronidazole) | yes |
| BAL 23 |  |  | No | yes |
| BAL 33 | [ - - - - - - - - ]  [ - - - - - - - - ] | **[*Ralstonia pickettii* (5 x 10^3^)]**  [***Lactobacillus oris* (10^4^)]** | No | yes |
| BAL 34 | [ - - - - - - - - ] | **[*Ralstonia pickettii* (5 x 10^4^)]** | Yes (amoxicillin-clavulanic acid) | yes |
| BAL 36 |  |  | Yes (cefotaxime and metronidazole) | yes |
| BAL 41 |  |  | Yes (piperacillin-tazobactam) | no |
| BAL 42 | [*Staphylococcus warneri* (< 10^3^)] | [ - - - - - - - - ] | No | no |
| BAL 43 | [ - - - - - - - - ] | **[*Streptococcus thermophilus* (8 x 10^6^)]** | Yes (cefotaxime and metronidazole) | yes |
| BAL 46 | [*Lactobacillus rhamnosus* (< 10^4^)] | [*Lactobacillus rhamnosus* (2 x 10^3^)] | Yes (cefotaxime and metronidazole) | no |

Comparison of the results of bacterial quantification by culture and mNGS in 32 BALFs. Only species identified by culture (present above or below the clinical threshold of 10^4^ CFU/mL) and/or those exceeding metagenomics threshold (5.3 x 10^3^ GEq/mL) in the mNGS analysis are presented. In bold: pathogen detections above the clinical threshold for culture or above the mNGS positivity threshold. Species not belonging to the pneumonia panel are indicated between square brackets. Dotted lines indicate no detection. False positive (FP) and true positive (TP) SOI (pneumonia panel) are indicated for mNGS using culture data as a reference. NI, non-interpretable quantification of SOI; indicated only for SOIs identified by culture (below or above clinical threshold). FP* and NI*: corresponding SOIs were detected by culture under the clinical threshold. “> MT” means that SOI was detected but SPC was undetected (see methods).

^a^ *belong to alpha-hemolytic streptococci*
